# From laboratory to clinical practice: an update of the immunological and molecular tools for neurocysticercosis diagnosis

**DOI:** 10.3389/fpara.2024.1394089

**Published:** 2024-07-01

**Authors:** Luz M. Toribio, Javier A. Bustos, Hector H. Garcia

**Affiliations:** ^1^ Infection and Immunity Institute, St George’s University of London, London, United Kingdom; ^2^ Center for Global Health, Universidad Peruana Cayetano Heredia, Lima, Peru; ^3^ Cysticercosis Unit, Instituto Nacional de Ciencias Neurologicas, Lima, Peru; ^4^ Center for Global Health, School of Sciences, Universidad Peruana de Ciencias Aplicadas (UPC), Lima, Peru; ^5^ Department of International Health, Bloomberg School of Public Health, Johns Hopkins University, Baltimore, MD, United States

**Keywords:** neurocysticercosis, immunodiagnosis, molecular diagnosis, *Taenia solium*, Peru

## Abstract

Neurocysticercosis (NCC) is caused by the invasion of *Taenia solium* larvae in the central nervous system (CNS) and stands as the predominant cause of epilepsy and other neurological disorders in many developing nations. NCC diagnosis is challenging because it relies on brain imaging exams (CT or MRI), which are poorly available in endemic rural or resource-limited areas. Moreover, some NCC cases cannot be easily detected by imaging, leading to inconclusive results. Multiple laboratory assays, principally immunological, have been developed to support the diagnosis and/or monitor the treatment efficacy, but its production can be costly, laborious, and non-globally accessible because they depend on parasite material. Therefore, recent advances have been focused on the implementation of recombinant or synthetic antigens as well as monoclonal antibodies for NCC immunodiagnosis purposes. Similarly, molecular diagnosis has been explored, obtaining promising results. Here we described the recent progress in the development of immunological and molecular diagnostic tools for NCC diagnosis over the past 13 years, discussing their potential application to address important challenges and how to focus future directions to improve NCC diagnosis with emphasis on enhance accessibility and the importance of test validation to provide an adequate support for clinical decisions.

## Introduction

Neurocysticercosis (NCC) is a neglected tropical disease resulting from the ingestion of *Taenia solium* eggs that can pass through the intestines, enter the bloodstream, and establish in the central nervous system (CNS), forming a cyst or cysticercus (Dixon et al., 2021). Its life cycle included humans and pigs as intermediate hosts, harboring the larvae or cysticercus, and humans as the definitive hosts for the tapeworm. This infection has public health significance, being the main contributor to acquired epilepsy and various others neurological morbidities worldwide (Cárdenas et al., 2021). The prevalence of NCC is not only high in all regions of the developing world but also there is an increasing frequency in industrialized countries due to immigration from endemic zones (Butala et al., 2021; Haddad et al., 2023).

This heterogeneous disease is typically asymptomatic during parasite establishment and when the cysts remain viable; however, the natural disease progression (or induced by anti-helminthic treatment) produces a degeneration process, resulting in an inflammatory reaction (Garcia et al., 2014). Clinical manifestations and host immunological responses are presented based on the stage, number, size, and principally the localization of cysts (Butala et al., 2021; Pineda-Reyes and White, 2022). Parasitic lesions that develop within the brain parenchyma (parenchymal NCC) typically transforms from viable, inflamed cysts to ultimately form calcified scars. Seizures are the primary clinical manifestation due to the focal inflammation of lesions, and prognosis is generally favorable (Del Brutto, 2022). On the other hand, extraparenchymal NCC involves the parasite invading the ventricles or subarachnoid space (subarachnoid NCC, SANCC) and growing massively, causing intracranial hypertension, and being associated to significant mortality rates (Mahale et al., 2015; Abanto et al., 2021).

The confirmation of NCC diagnosis absolutely requires neuroimaging; however, immunodiagnostic tests are accessible diagnostic tools that support imaging findings, especially in rural or resource-limited areas (Del Brutto, 2012). The host immune response has been well studied over the years, identifying a general pattern of immunodominance based on NCC classification. SANCC patients are the most immunodominant (Nash and O’Connell, 2020), followed by parenchymal with viable multiple cysts. Immunodominance decreases depending on the number of cysts (Garcia et al., 2018a; Channel et al., 2021), with single lesions being generally undiagnosed by common immunological tests. Finally, patients with calcified lesions have fewer detectable immune responses (Nash et al., 2015; Nash et al., 2017a; Nash et al., 2017b). In this context, immunodiagnostic tools based on antibody and/or antigen detection could help to discriminate viable and/or severe infections for a confirmatory diagnosis and follow-up (Deckers and Dorny, 2010; Zea-Vera et al., 2013).

Over the years, various diagnostic tools for NCC have been developed and tested, but not all have been properly implemented. As members of the Cysticercosis Working Group in Peru, we have compiled this manuscript to summarize our most recent contributions to the development of laboratory tests and have additionally included some published works from other groups. Although we are aware that the immunological response is not only driven by the parasite’s establishment in the brain but also by muscular cysticercosis, we have focused this review on published advances in diagnostic techniques only tested for NCC to simplify the understanding of this complex disease. The primary aim of this review is to update the current knowledge about new immunological and molecular techniques and propose future applications for them, providing solutions to challenging scenarios in NCC diagnosis.

## Traditional immunodiagnostic tools

The definitive diagnostic test for NCC is neuroimaging; however, its use is limited. Immunological tests to detect antibodies and antigens have been widely used as a support for diagnosis (Gilman, 2016; Del Brutto, 2022). They were initially developed for cerebrospinal fluid (CSF) samples and now are commonly used in serum, which is relatively easier to obtain.

Antibody detection using the test of choice, enzyme-linked immunoelectrotransfer blot (EITB) (Tsang et al., 1989) or western blot, is based on a mix of seven glycoproteins purified from *T. solium* cysts, offering an excellent performance (100% sensitivity and specificity) (Tsang et al., 1991). However, the purification process demands substantial amounts of parasite material, which is difficult to obtain, and the purification technique is costly and laborious. Studies have demonstrated that antibody levels become detectable at 4 weeks after infection and typically persist for many years after the infection has resolved (Garcia et al., 2001; Deckers et al., 2008), making it unsuitable for early diagnosis or as an indicator of an active infection. On the other hand, parasite antigen can be detected as soon as 2 weeks after infection (Arroyo et al., 2022a) and confirms the presence of live parasites, providing an immunological tool for discriminate active infections (Zea-Vera et al., 2013). However, sensitivity is usually lower than antibody test (90% versus 100%), and its detection relies on monoclonal antibodies (moabs) that are only genus-specific and cross-react with other *Taenia* species (Garcia et al., 1998; Bustos et al., 2019).

Despite these drawbacks, decades of study have positioned these two serological tests as the principal tools to support NCC diagnosis, and the interpretation of results has been studied in detail in different clinical scenarios (Parija and Gireesh, 2011). These serological tests are described in detail below.

### Traditional antibody detection and antibody patterns

Currently, EITB (Tsang et al., 1989) is the serological gold standard for NCC. The EITB detects specific antibodies against seven lentil-lectin purified glycoproteins (LLGP) named according to their molecular weight: GP50, GP42–39, GP24, GP21, GP18, GP14, and GP13. This test has an exceptional performance, nearly 100%, for more than two viable lesions, with reduced sensitivity in single or calcified lesions (Garcia et al., 2018a). Although a positive result can be determined by the response against any of these glycoproteins, diagnostic LLGP antigens used for antibody detection have specific functions and are classified into three protein families: GP50, T24, and 8 kDa. The GP50 family (GP50 band) is a glycosylphosphatidylinositol (GPI) related to membrane-anchoring function. The T24 family (GP42–39 and GP24 bands) is a transmembrane tetraspanin participating in processes of cell proliferation, adhesion, motility, and scaffolding. Lastly, the 8 kDa family (low molecular weight bands GP21, GP18, GP14, and GP13) consists of hydrophilic proteins lacking transmembrane regions, suggesting their involvement in secretion activity and evasion of the host immune response (Hancock et al., 2003; Hancock et al., 2004; Hancock et al., 2006; Rodriguez et al., 2012). Some of these small antigens from the 8 kDa family have demonstrated to share epitopes with antigenic regions of the T24 family (Rodriguez et al., 2012).

**Figure 1:**
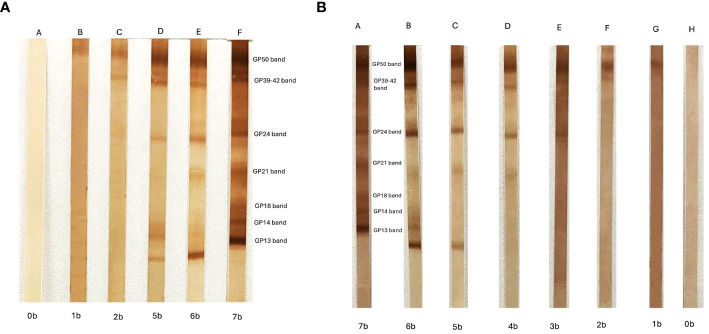
**(A)** Enzyme-linked immunoelectrotransfer blot (EITB) appearance antibody pattern obtained using consecutive serum samples from a pig experimentally infected with cysticercosis. The position of the seven antibody bands and the names are shown. “A” indicates the basal EITB result (0b = 0 bands) before the infection, and “B” to “F” are the EITB results during the time that the pig was followed (5 months after infection). **(B)** EITB disappearance antibody pattern obtained using consecutive serum samples from a neurocysticercosis patient under antiparasitic treatment. “A” indicates the initial EITB result (7b = 7 bands), and “B” to “H” are the EITB results during the time that the patient was under treatment and the infection was finally resolved.

The natural progression of NCC infection involves different antibody responses represented as antibody banding patterns in the EITB. These patterns have been identified in clinical patients and animal models and associated to the cyst stage (viable or degenerated) (Arroyo et al., 2018). The study of the order of antibody appearance (or disappearance) has shown that, during the course of infection, the antibody pattern usually appears in a consecutive order, starting with the heaviest protein GP50 band and concluding with the smallest GP13 band (Deckers et al., 2008). The antibody pattern of disappearance during the cyst degeneration process could take years and occurs in the opposite order (from GP13 to GP50) (Garcia et al., 2018a) (**Figure 1**). When we studied the responses against each family individually, we showed that antibodies against GP50 are the first to appear and last the longest; these are present since exposure or at the initial stages and maintained after cyst resolution. Responses against the T24 family are heterogeneous. A strong immunodominance is characteristic of this protein family, allowing antibodies to persist for many years, as GP50; however, they can also be associated with viable and extraparenchymal infections. Responses against any band from the 8 kDa family are commonly related to active infections or high antigen burden (multiple or extraparenchymal NCC), being the first to disappear after cyst resolution (Garcia et al., 2012; Rodriguez et al., 2012; Arroyo et al., 2018).

In conclusion, these strong associations between specific antibody responses and clinical aspects of the disease suggest that a careful interpretation of antibody patterns can provide important serological information for the diagnosis, characterization, and follow-up of NCC cases.

### Traditional antigen detection

Another widely studied immunological test is the direct detection of parasite antigens using the antigen-enzyme-linked immunosorbent assay (Ag-ELISA). The Ag-ELISA represents a valuable tool for NCC diagnosis and follow-up as antigen levels are strongly associated with viability, parasite burden, and decrease rapidly after successful antiparasitic treatment (Deckers and Dorny, 2010; Rodriguez et al., 2012). The first Ag-ELISA based on monoclonal antibodies was initially developed for CSF samples using HP10 which used IgM moabs against a repetitive epitope from an excretory/secretory glycoprotein of *Taenia saginata* cysts, which cross-reacted with *T. solium* cysticercosis infection. Later, the performance in serum samples was compared to CSF, demonstrating to be similar by detecting between 96.5% to 80% of viable lesions and 40% of calcified lesions, with specificity of 94% (Fleury et al., 2013; Parkhouse et al., 2018).

Subsequently, the implementation of the B158/B60 Ag-ELISA significantly improved antigen detection. This test also relied on IgM moabs B158/B60 against an excretory/secretory product of 65 kDa from *T. saginata* metacestode (Draelants et al., 1995; Jansen et al., 2016). The B158/B60 Ag-ELISA was optimized by changing the moabs isotype to IgG, which resulted in overall sensitivity of 90% for living cysts and specificity of 98.7% for NCC (Dorny et al., 2003; Rodriguez et al., 2009; Gómez-Morales et al., 2017). The B158/B60 Ag-ELISA quickly emerged as the preferred and most widely used assay for antigen detection diagnosis in NCC, leading to the development of a commercial version [Cysticercosis Ag ELISA, ApDia, Belgium) with sensitivity of 98% (ELISA cysticercosis ag kit (REF.650501)]. Although these moabs are only genus-specific and cross-reacted with other *Taenia* species, it is a useful tool for the diagnosis of NCC since *T. solium* is the only species causing human cysticercosis (Castillo et al., 2023).

Many studies have focused on the association of antigen levels in NCC patients with different characteristics and clinical spectrums. Elevated antigen levels are associated with extraparenchymal (SANCC), viable multiple NCC, and patients with epilepsy, while negative or low antigen levels are found in calcified or single lesions (Gabriël et al., 2012; Garcia et al., 2012). Additionally, another important use of Ag-ELISA is for monitoring of treatment efficacy. The short longevity of released antigens results in a gradual decrease in antigen levels after antiparasitic treatment, serving as a predictor for successful therapy (Fleury et al., 2013; Rodríguez-Hidalgo et al., 2019). Therefore, measuring antigen levels by Ag-ELISA has been crucial in support diagnosis when the imaging results are unclear and has shown as a promising candidate for monitoring treatment efficacy.

## Latest advances in immunological tests

### Antibody detection

#### Development of recombinant antigens (derived from EITB)

EITB glycoproteins have been fully characterized, but their limited availability restricts their use. We have characterized and cloned homologous proteins and synthetic peptides of the three EITB diagnostic protein families (rGP50, rT24H, and peptides sTsRS1, sTs18var1, sTsRS2var1, and sTs14) (Greene et al., 2000; Hancock et al., 2003; Hancock et al., 2004; Hancock et al., 2006), providing advantages in accessibility and reproducibility for measuring specific antibody responses. These have been evaluated to demonstrate comparable performance to their native counterparts in the EITB (sensitivity 90%–100% and specificity 93%–100% for more than two viable cysts) (Handali et al., 2004; Bueno et al., 2005; Handali et al., 2010a; Lee et al., 2011a). A detailed review of the production and application of these glycoproteins was conducted in 2012 (Rodriguez et al., 2012), and since then, additional studies have developed new tools focusing on antigen rT24H. An ACEK POC immunosensor test (Liang et al., 2019), rT24H EITB (Dermauw et al., 2018; Stelzle et al., 2023), ELISA (Lee et al., 2011b), and the validation of an rT24H lateral flow assay (Handali et al., 2010b; Corstjens et al., 2014) have been reported.

As previously mentioned, the study of antibody patterns may enhance the understanding of the immune responses during NCC infection (Praet et al., 2010; Arroyo et al., 2022b). Therefore, simultaneous antibody response tests have also been developed, such as an immunoblot based on rGP50, rT24H, and sTsRS1 (Noh et al., 2014) and two multiantigen printing immunoassays (MAPIA) (Handali et al., 2010c; Toribio et al., 2023b), obtaining acceptable performance in viable and extraparenchymal NCC (over 90% of sensitivity). The easy handling and assembling of MAPIA represents an important advantage for large-scale assay production. The first MAPIA (Handali et al., 2010c) compared the performance of all six recombinant proteins, reaching 97% and 99% of sensitivity and specificity for patients with intra- and extra-parenchymal NCC. However, it was concluded that using all six recombinant antigens did not improve its sensitivity. More recently and highlighting the advantage that these proteins are now released from patent rights, an optimized MAPIA using only three antigens (rGP50, rT24H, and sTs14) of each protein diagnostic family was developed (Toribio et al., 2023b). It detected 100% of SANCC and 97.5% of parenchymal cases. Another approach based on the quantitative measurement of antibody responses is the development of a multiple triplex ELISA using rGP50, rT24H, and sTs18var3 (Tang et al., 2023), with an excellent concordance with the EITB (over 98% agreement), and a multiplex bead assay (MBA) using two forms of rT24H and recombinant Ts8B2 that showed high sensitivity in cases with two or more viable cysts (sensitivity of 96.1% and specificity of 96.5%) (Hernández-González et al., 2017; Moss et al., 2018).

A detailed study of the antibody responses against recombinant antigens in different NCC scenarios is needed to prove their potential contribution in diagnosis. In fact, measuring different antibody responses simultaneously and quantifying them should contribute enormously to the understanding of antibody pattern interpretation.

#### Other tools for antibody detection

Some commercial kits for serum antibody detection have been developed (DRG™, RIDASCREEN™, NOVATECH™, CYPRESS™, and IVD™, among others) using native or less purified versions of total and vesicular antigen of metacestodes. Although they offer quantitative and rapid results, they have suboptimal sensitivity (under 72%) and specificity (~60%) (Gómez-Morales et al., 2017; Zammarchi et al., 2018; Hernández et al., 2019). One study that tested RIDASCREEN™ and NOVATECH™ showed a sensitivity rate lower than 44% in viable parenchymal lesions and <6.7% for calcified NCC and a specificity rate between 52.2% and 71.1%. Additionally, cross-reaction with hydatic disease and hymenolepiasis was confirmed (84.4% and 11.1%, respectively) (Chuang et al., 2017; Garcia et al., 2018b).

Various other purified and recombinant antigens have been developed in the past 13 years. These include crude soluble extract (Arora et al., 2020), purified antigens [others LLGP version (Ayala-Sulca and Miranda-Ulloa, 2015; Davelois et al., 2016), ag 52–53 kDa (Piña et al., 2011), cathepsin L (León-Janampa et al., 2019), TsAg5 (Rueda et al., 2011), LLa-Gp (Suzuki and Rossi, 2011), and Jacalin purified (MaChado et al., 2013)], excretory/secretory antigens, and lower molecular mass (10–30 kDa) (Atluri et al., 2009; Sako et al., 2015) using ELISA, Dot ELISA, and immunoblot techniques, but which have not been validated for its use in diagnosis. One of the most promising tests uses the recombinant Tsol-p27 with sensitivity above 85% and specificity of ~96% (Salazar-Anton et al., 2012; Nhancupe et al., 2016; Nhancupe et al., 2019). However, its initial description was not followed by further performance assessments. A detailed description of the developed immunological tests is shown in **Table 1A**.

**Table 1A:** Principal immunological tests assessed in neurocysticercosis (NCC) patients for antibody and antigen detection in the last 13 years.

Test name	Year	Source	Sample	Reported use	Sensitivity	Specificity
Antibody detection						
1. Total or semipurified antigens						
ELISA: Enzitest^®^ IgG (Carvalho Junior et al., 2010)	2010	Antigens from metacestode	CSF	Tested in clinical NCC samples	31.80%	100%
ELISA and EITB (Parija and Raman, 2011)	2011	Purified antigen	Serum	All patients with epilepsy	*	*
Lla-Gp ELISA (Suzuki and Rossi, 2011)	2011	Lla-Gp fraction	Serum	Tested in clinical NCC samples	95.50%	100%
Dot ELISA (Piña et al., 2011)	2011	Cathepsin-L	Serum	Tested in clinical NCC samples: EP, MP, S	94.4%, 74.6% and 29.4%	99-100%
ELISA DJ unbound (MaChado et al., 2013)	2013	Jacalin unbound antigen fraction purified with detergent (DJ unbound)	Serum	Tested in clinical NCC samples	93%	93.50%
DEAE ELISA (Ribeiro Vda et al., 2014)	2014	Diethylaminoethyl binding fractionS (DEAE)	Serum	Tested in clinical NCC samples	90%	90.40%
TsM PS ELISA (Pappala et al., 2017)	2017	*T. solium* protoescoleces antigens	Serum	Tested in clinical NCC samples: single and multiple lesions	86.60%	98.30%
Cyst fluid EITB (Arora et al., 2020)	2020	Cyst fluid purification by ultracentrifugation (antigen 15 kDa)	Serum	Community-based trial	91.50%	91.60%
Cysticercosis fluid ICT POC-test (Sadaow et al., 2023)	2023	Antigens from cysticercosis fluid	Serum	Tested in clinical NCC samples	83.30%	92%
2. LLGP recombinant and synthetic peptides						
MAPIA (Handali et al., 2010c; Toribio et al., 2023b)	2010	rGP50, rT24H, sTs14, sTs18var1, sTsRS1, sTsRS2var1	Serum	Tested in clinical NCC samples: >2 lesions	97%	99.40%
	2023	rGP50, rT24H, and sTs14	Serum	Tested in clinical NCC samples: EP, P	100% EP NCC, 97.5% parenchymal NCC	98.53%
Tsol-p27 immunoblot (Salazar-Anton et al., 2012; Nhancupe et al., 2016)	2012	Recombinant Tsol-p27	Serum	Tested in clinical NCC samples	86.70%	97.80%
	2016	Recombinant Tsol-p27	Serum	Tested in clinical NCC samples	85.71%	96.69%
Recombinant EITB (Noh et al., 2014; Dermauw et al., 2018)	2014	rGP50, rT24H, and sTsRS1	serum	Tested in clinical NCC samples	99%	99%
	2018	rT24H	Serum	Tested in clinical NCC samples: >2 lesions	80%	98.2%
rT24H lateral flow (POC assay) (Lee et al., 2019; Mubanga et al., 2021; Stelzle et al., 2023)	2019	rT24H	Serum	Tested in clinical NCC samples: EP, MP, S	89%	99%
	2021	rT24H	Serum	Community-based trial	35%	87%
	2023	rT24H	Serum	Community-based trial	49%, 98% only viable	*
Multiplex GST-Ts8B, GST-T24H, and T24H-his (Hernández-González et al., 2017; Moss et al., 2018)	2017	Recombinant GST-Ts8B, GST-T24H, and T24H-his	Serum	Tested in clinical NCC samples: single and multiple lesions	96.10%	96.50%
	2018	Recombinant GST-T24H	Serum	Population screening in endemic regions	Prevalence 8%	
Triplex ELISA (Tang et al., 2023)	2023	rGP50, rT24H, and sTs18var3	Serum	Tested in clinical NCC samples: EP, P	98% concordant with LLGP-EITB	
Antigen detection						
HP10 Ag-ELISA (Fleury et al., 2013; Parkhouse et al., 2018)	2013	moabs HP10	Serum	Follow-up: EP	*	*
	2018	moabs HP10	CSF and serum	Tested in clinical NCC samples: EP	95.3% CSF and 52.3% serum	*
scFv Ag-ELISA (Ribeiro Vda et al., 2013)	2013	Single chain variable fragment (scFv) antibodies	Serum	Tested in clinical NCC samples	97%	95%
IgY ELISA (Carrara et al., 2020)	2020	IgY from immunized hens	Serum	Tested in clinical NCC samples	93.20%	94.30%
TsG10 ELISA (Corda et al., 2022)	2022	Recombinant moabs TsW	Serum	Tested in clinical NCC samples: EP	98%	100%
TsW8/TsW5 Ag-ELISA (Castillo et al., 2023)	2023	moabs TsW8/TsW5	Serum	Tested in clinical NCC samples: EP, P, C	97.8% EP NCC, 79.2% parenchymal NCC, 43.9% calcified NCC	*
TsW8/TsW5 POC Test (Toribio et al., 2023a)	2023	moabs TsW8/TsW5	Urine	Tested in clinical NCC samples: EP	97%	100%

EP, extraparenchymal NCC; P, parenchymal NCC; MP, multiple parenchymal lesions; S, single lesion; C, calcified NCC; *, not assessed.

**Table 1B:** Principal molecular tests assessed in neurocysticercosis (NCC) patients in the last 13 years.

Test name	Year	Target	Nature	Sample	Used	Sensitivity	Specificity
PCR (Almeida, 2006)	2011	pTsol9	Repetitive sequence	CSF	Tested in clinical NCC samples	95.90%	80%–100%
PCR (Roelfsema et al., 2016)	2016	12S	Mitochondrial	Parasite material	Identification of species	*	*
PCR (Toribio et al., 2019)	2019	pTsol9	Repetitive sequence	Urine	Tested in clinical NCC samples: EP, P, C	83% viable, 74% overall	98%
PCR (Goyal et al., 2020a)	2020	pTsol9	Repetitive sequence	Blood, urine	Community-based trial	57% blood, 64% urine	94% blood, 87% urine
LAMP (Goyal et al., 2020b)	2020	cox1	Mitochondrial	Blood	Tested in clinical samples: EP, P	71.8% P, 86.7% EP	90%
q-PCR (O’Connell et al., 2020)	2020	TsolR13	Repetitive sequence	CSF, plasma	Tested in clinical samples: EP	100% CSF, 81.3% plasma	100%

EP, extraparenchymal NCC; P, parenchymal NCC; C, calcified NCC; *, not assessed.

### Antigen detection

#### Current use and validation of B158/B60 Ag-ELISA

In recent years, the reference test B158/B60 Ag-ELISA has been further studied and validated (Jansen et al., 2016) and extensively employed for routine clinical diagnosis and epidemiological studies (Moyano et al., 2014; Carod et al., 2021). Antigen detection with B158/B60 moabs has been improved using urine samples, providing a significant advantage as it is non-invasive.

One of the first reports in urine was conducted by our group in 2009 (Castillo et al., 2009), obtaining a sensitivity of 92% for viable intra- and extraparenchymal NCC. As expected, sensitivity decreased to 62.5% for patients with a single lesion and to 16.7% for calcified NCC. Since then, B158/B60 Ag-ELISA in urine has been used as a screening test, principally for SANCC detection in endemic communities. The positive predictive value for this ELISA was assessed as being 62% for SANCC and patients with high antigen levels (Ag OD ratio >3), also demonstrating that low antigen levels are poor predictors of SANCC (Mwape et al., 2011; Garvey et al., 2018; McCleery et al., 2020). The usefulness of antigen detection in urine for the early identification of NCC cases needs to be further studied (Sahu et al., 2014).

#### Development of new monoclonal antibodies

The use of monoclonal antibodies (moabs) for antigen detection was initially performed using HP10 moabs, followed by B158/B60 moabs, both produced against *T. saginata*. After decades, new *T. solium* moabs have been developed by our group. A set of 21 clones of moabs against whole cyst (TsW1–13), vesicular fluid (TsV1–5), and excretory/secretory antigens (TsE1–3) was developed, and eight of them were able to detect antigens in body fluids (CSF, serum, and urine). A novel Ag-ELISA was later standardized using moabs TsW8 as the capture antibody and TsW5 as the detection antibody, both directed against whole cyst antigens. The initial report showed a cross-reaction of these moabs with *T. saginata* antigens (Paredes et al., 2016). Although none of them were reactive against *Fasciola hepatica*, *Echinococcus granulosus*, and *Taenia hydatigena*, a recent study reported the cross-reaction of *T. hydatigena* antigens in pigs (Paredes et al., 2016; Arroyo et al., 2024).

Recently, one study (Castillo et al., 2023) compared TsW8/TsW5 Ag-ELISA performance against the B158/B60 Ag-ELISA. Both assays demonstrated high levels of agreement (over 97%) in serum from patients with subarachnoid, parenchymal, and calcified NCC, suggesting a new role for TsW8/TsW5 in accurately detecting antigen levels in serum samples. Moreover, ongoing studies are currently assessing the utility of TsW8/TsW5 Ag-ELISA to accurately detect antigen in serum and in urine.

#### Other tools for antigen detection

Nanobodies have been established as promising tools for antigen detection due to their easy handling and high stability. However, only one report (2009) about nanobodies against an 8-kDa antigen (sTs14) was published (Deckers et al., 2009), and no further evaluations have been conducted since then. Single-chain variable fragment (scFv), produced by phage display against *T. solium* total saline extraction, has been reported (Ribeiro Vda et al., 2013). The three scFv antibodies (G10, A4, and B6) presented a sensitivity rate of 97% for NCC patients (NCC type was not mentioned) and a specificity rate of 95%. Similarly, a novel recombinant moab (TsG10) has been developed from previous IgM-secreting hybridomas (Paredes et al., 2016); its use could decrease the cost of moabs production (Corda et al., 2022). Performance was assessed in serum, plasma, and CSF from patients with extraparenchymal NCC, achieving a sensitivity rate of 98% and a specificity rate of 100%. These promising results may encourage the use of these more affordable antibody options to detect circulating antigen.

Finally, the use of TsW8/TsW5 moabs in urine in a point-of-care (POC) test format detected 97% of extraparenchymal NCC patients with a specificity rate of 100% (Toribio et al., 2023a). This rapid test can be performed in 15 min in urine samples and constitutes a valuable tool for rural settings. Studies are being carried out to determine the performance of this rapid test and to validate its use as a screening tool in low-resource areas.

## Latest advances in molecular tests

Since the introduction of the PCR assay, molecular diagnosis has assumed a crucial role in diagnosing diseases where conventional immunological tests fall short. PCR offers significant advantages over immunological tests, increasing its sensitivity and specificity by amplifying very low quantities of specific parasite DNA sequences (Li et al., 2011). Several PCR tests have been developed for the detection of *T. solium* DNA, primarily for phylogeny and taxonomy analysis or its direct use for confirming *T. solium* infection in pig carcasses (Ramahefarisoa et al., 2010; Sreedevi et al., 2012; Gauci et al., 2019; Satyaprakash et al., 2023).

More recently, the detection of circulating cell-free parasite DNA fragments (cfDNA) has been explored. cfDNA comprises small nuclear or mitochondrial DNA fragments released from cells into body fluids; the origin and mechanisms of DNA release are not fully understood but are associated with cell death pathways and active secretion. cfDNA molecules are released as small fragments from 50 to 400 bp in urine, after they have been cleared in the kidney by the glomerular barrier (Toribio et al., 2020; Diao et al., 2022; Lozano et al., 2023). Detection of cfDNA offers a convenient sample handling due to its stability and, more importantly, eliminates the dependence on parasite material for its development by using commercially synthetic primers.

In NCC diagnosis, cfDNA was first detected in CSF, recognizing a repetitive sequence of the *T. solium* genome (pTsol9) in 29/30 NCC cases (95.9%), including parenchymal, subarachnoid, and calcified lesions (Almeida et al., 2006). Subsequent studies developed other PCR assays targeting the same (Toribio et al., 2019; Goyal et al., 2020a) or different sequences (Michelet et al., 2011) in CSF, blood, and urine samples. Besides pTsol9 sequence, another promising target is a repetitive sequence (TsolR13), which was tested in a quantitative assay (qPCR) obtaining a sensitivity rate of 100% for extraparenchymal NCC diagnosis (Michelet et al., 2011; O’Connell et al., 2020). Moreover, one POC-test based on loop-isothermal amplification (LAMP) directed against mitochondrial gene COX1 was also developed, obtaining a sensitivity rate of 71.8% and 86.7% for parenchymal and extraparenchymal NCC, respectively (see **Table 1B**) (Goyal et al., 2020b).

## Discussion

This literature review of new immunological and molecular diagnostic tools is focused only on NCC diagnosis. As members of one of the most productive cysticercosis research groups, we have explained in detail our contributions, as well as some others, to the NCC diagnosis. Major advances in clinical laboratory diagnostic technologies (development of recombinant antigens, new monoclonal antibodies, and the exploration of the molecular diagnosis) have resulted in high-quality, safe, and effective clinical decision tools. However, NCC diagnosis continues to face challenges, including its limited accessibility in many scenarios (Mlowe et al., 2022). In this final section, we propose possible solutions for diagnostic challenges using new tests and future lines of research.

### Challenges and current perspectives

NCC diagnosis is challenging due to the clinical heterogeneity of the immunological response and the variability of antigen/antibody expression during different stages of the disease (Ito, 2013; Ostoa-Saloma et al., 2013; Hossain et al., 2023). Additionally, available diagnostic tests are limited by their low sensitivity in some NCC forms and difficult accessibility (Mlowe et al., 2022; Takayanagui and Haes, 2022). Here we propose the use of new laboratory tests to address these specific scenarios of NCC:

#### Single brain NCC lesions

These lesions represent a major challenge in diagnosis due to two principal reasons: neuroimaging commonly confuses them with other pathologies, and they are underdiagnosed by the traditional immunological assays because of their low levels of antigen/antibody responses. More sensitive assays can contribute to improve single lesion detection. One strategy to address this challenge is the simultaneous detection of various antibodies to enhance the sensitivity using our new MAPIA with higher antigen concentrations of recombinant antigens to capture lower levels of antibody responses or by taking advantage of the high capacity of beads to capture antibodies in their spherical surface used in the triplex ELISA or the multiplex bead assay (Hernández-González et al., 2022).

Another alternative recently explored is the use of gene expression from monocytes to differentiate NCC single parenchymal lesion from brain tuberculomas. The expression of NCC-associated monocyte genes (TAX1BP1, RAP1A, PLCG2, TOR3A, GBP1P1, LRRFIP2, and FEZ2) has a sensitivity rate of 87.2% and a specificity rate of 100% (combination of TAX1BP1-RAP1A and GBP1P1 genes) in single lesions (Pamela et al., 2021).

#### Extraparenchymal NCC

A prompt detection of extraparenchymal NCC cases, especially SANCC, is crucial, as it is the most severe form of NCC and the one with the worst prognosis. Recent studies in endemic areas of Peru have detected an unexpectedly higher prevalence of SANCC in asymptomatic villagers (Mahale et al., 2015). Priority should be its rapid identification through practical immunological tests, followed by imaging diagnosis for confirmation to provide an adequate clinical management. Since these patients release great quantities of antigens into circulation, the approach would be the use of a reliable and practical test with high specificity, capable of discriminating active forms of this severe case of NCC to be applied as a screening test especially in rural settings. The Ag-ELISA in urine can be useful (Gabriël et al., 2012) to determine antigen levels in a quantitative manner; however, for mass screening, interventions may result as impractical and costly. Our new POC assay, based on moabs TsW8/TsW5, could also serve as an affordable and practical tool to quickly identify active SANCC cases in non-invasive samples to be later referred for imaging confirmation and specialized care.

#### Monitoring treatment efficacy

Post-treatment follow-up is essential to assess the effectiveness of antiparasitic treatment and make clinical decisions. Ideally, neuroimaging studies would be employed for this purpose, but they are expensive and logistically impractical (Hamamoto Filho et al., 2022). The strategy here should be the use of quantitative tests, such as Triplex ELISA, Ag-ELISA (Zea-Vera et al., 2013; Rodríguez-Hidalgo et al., 2019) or multiplex beads assay to evaluate antigen/antibody levels in samples collected from patients undergoing antiparasitic treatment. Urine collection is non-invasive and painless to collect, providing an easy-to-access sample. Studying the antigen/antibody dynamics in response to treatment and a simultaneous identification of one or a set of biomarkers that rapidly change in response to alterations in parasite viability could define an earlier time point for confirming treatment success, reducing costs and the period of uncertainty for patients under treatment (Hamamoto Filho et al., 2022). Lastly, quantitative PCR has been reported for the detection and follow-up of NCC patients, constituting a possible new tool for monitoring treatment efficacy (Rottbeck et al., 2013).

Regarding new molecular diagnostic tests, exploring the potential of cfDNA detection could be promising to support NCC diagnosis in specific scenarios and for monitoring treatment efficacy. DNA amplification should increase the sensitivity, making it a good candidate to detect single lesions with lower antibody/antigen. Moreover, detection in non-invasive samples, the easy handling of cfDNA, and accessible synthesis for its development could benefit a screening application. The limitations of DNA assays need to be further explored; we anticipate that could be the same as immunological assays, being the difficulty to differentiate muscular cysticercosis from NCC since cfDNA is actively released from cysts in any location. DNA technologies are usually expensive, limiting its application in a large scale. However, some rapid and affordable techniques have been mentioned (such as LAMP). In addition, some reports have demonstrated the possibility to detect DNA in a significant proportion of calcified NCC cases using PCR (around 40%), not allowing an adequate discrimination of active and inactive NCC cases.

### Future directions

Future directions for NCC diagnosis should focus on enhancing the accessibility, reliability, and efficiency of current and new diagnostic tools. An adequate validation of methods is critical to ensure their effectiveness in different scenarios and to help the clinical practice (Gjorup, 1997; Ito, 2013; Cardona-Arias et al., 2017).

Accessibility to immunological tests was improved with the use of recombinant forms of the EITB glycoproteins (rGP50 and rT24, sTSRS1, sTS18var1, sTS18var1, sTSRS2var1, and sTs14); their easier production reduces costs and improves assay reproducibility. Molecular diagnosis based on synthetic primers can also be accessible for any laboratory. Hence, its use should be exploited and applied to more studies (Esquivel-Velázquez et al., 2011).

The reliability of an assay is determined by a combination of factors, including accuracy, precision, consistency, sensitivity, specificity, and reproducibility (Bolarinwa, 2015; Gómez-Morales et al., 2017; Ramachandran and Wilson, 2018). Methods for evaluating this depend on the assay type and the analyte, but general guidelines can be followed. To evaluate accuracy, the use of reference standards and controls with known values (or concentrations) must be implemented. Precision can be assessed by measuring inter- and intra-assay variations (Manterola et al., 2018). In the same way, consistency and robustness are evaluated by determining the tolerance of the test to environmental variations (temperature, pH, and others). Finally, reproducibility can be ensured through participation in collaborative studies or ring trials with other institutions (Jansen et al., 2016; Gómez-Morales et al., 2021).

Assessing sensitivity and specificity is crucial to define test performance and for an adequate validation. The best way to determine them is evaluating a well-defined set of samples in a ROC curve analysis (Jayashi et al., 2014). Here we proposed a practical manner for defining NCC groups based on its immunological response (Garcia et al., 2014), including extraparenchymal (SANCC), multiple parenchymal with severe (>10 cysts), moderate (five to 10 cysts), mild (three to five cysts), low infection (one to three cysts), and single lesions. On the other hand, for specificity, a large group of appropriate negative controls must be tested. It is also important to mention that the establishment of the cutoff point should be flexible and aligned with the study aim—for example, for screening tests focused on the detection of severe cases with a high immunological response, specificity should be maximized, while for identifying single brain lesions, sensitivity must be prioritized over specificity.

With the diagnostic tools currently available and their standardized validation, it will be possible to implement one or more rapid, accurate, and worldwide-accessible diagnostic tests for the early and accurate detection of NCC, both in clinical and in field conditions, as well as tools for monitoring treatment success. It is clear that diagnosis cannot rely only on laboratory tests but must adequately support the imaging findings and guide the clinician for appropriate clinical decisions. A rigorous validation of new tests could facilitate the decision-making regarding the implementation (or not) of these tests in the laboratory, and a careful interpretation of results should be implemented to update the global scheme of diagnosis criteria.

## Author contributions

LT: Formal analysis, Investigation, Methodology, Project administration, Writing – original draft, Writing – review & editing. JB: Data curation, Resources, Supervision, Writing – original draft, Writing – review & editing. HG: Conceptualization, Data curation, Funding acquisition, Resources, Supervision, Writing – original draft, Writing – review & editing.
